# Advancing dental biofilm models: the integral role of pH in predicting *S. mutans* colonization

**DOI:** 10.1128/msphere.00743-24

**Published:** 2024-12-11

**Authors:** Jay S. Sangha, Valentina Gogulancea, Thomas P. Curtis, Nicholas S. Jakubovics, Paul Barrett, Aline Metris, Irina D. Ofiţeru

**Affiliations:** 1 School of Engineering, Newcastle University, Newcastle upon Tyne, United Kingdom; 2 Faculty of Medical Sciences, Newcastle University, School of Dental Sciences, Newcastle upon Tyne, United Kingdom; 3 Safety and Environmental Assurance Centre, Unilever, Bedfordshire, United Kingdom; NC State University, Raleigh, North Carolina, USA

**Keywords:** *Streptococcus mutans*, dental plaque, individual-based modeling, biofilm dynamics, hydroxyapatite coupons

## Abstract

Mathematical models can provide insights into complex interactions and dynamics within microbial communities to complement and extend experimental laboratory approaches. For dental biofilms, they can give a basis for evaluating biofilm growth or the transition from health to disease. We have developed mathematical models to simulate the transition toward a cariogenic microbial biofilm, modeled as the overgrowth of *Streptococcus mutans* within a five-species dental community. This work builds on experimental data from a continuous flow reactor with hydroxyapatite coupons for biofilm growth, in a chemically defined medium with varying concentrations of glucose and lactic acid. The biofilms formed on the coupons were simulated using individual-based models (IbMs), with bacterial growth modeled using experimentally measured kinetic parameters. The IbM assumes that the maximum theoretical growth yield for biomass is dependent on the local concentration of reactants and products, while the growth rates were described using traditional Monod equations. We have simulated all the conditions studied experimentally, considering different initial relative abundance of the five species, and also different initial clustering in the biofilm. The simulation results only reproduced the experimental dominance of *S. mutans* at high glucose concentration after we considered the species-specific effect of pH on growth rates. This highlights the significance of the aciduric property of *S. mutans* in the development of caries. Our study demonstrates the potential of combining *in vitro* and *in silico* studies to gain a new understanding of the factors that influence dental biofilm dynamics.

**IMPORTANCE:**

We have developed *in silico* models able to reproduce the relative abundance measured *in vitro* in the synthetic dental biofilm communities growing in a chemically defined medium. The advantage of this combination of *in vitro* and *in silico* models is that we can study the influence of one parameter at a time and aim for direct validation. Our work demonstrates the utility of individual-based models for simulating diverse conditions affecting dental biofilm scenarios, such as the frequency of glucose intake, sucrose pulsing, or integration of pathogenic or probiotic species. Although *in silico* models are reductionist approaches, they have the advantage of not being limited in the scenarios they can test by the ethical consideration of an *in vivo* system, thus significantly contributing to dental biofilm research.

## INTRODUCTION

Dental biofilms (also known as dental plaque) play an important role in oral health and disease, for example in the development of dental caries and periodontitis (1). The diversity of dental biofilms is vast, being the second largest microbial community in humans after the gut (2). It is consequently challenging, if not impossible, to disentangle *in vivo* the inherent complexity of interactions between hundreds of different species and how they are affected by external factors such as diet or oral hygiene.

Mathematical models have significantly contributed to the advancement of the biofilm research field (3). Moreover, when used together with *in vitro* models, *in silico* models can guide experiments and help formulate hypotheses that can be tested experimentally (4). However, the application of *in silico* modeling for dental biofilms has been relatively limited to date. The ground was set by the pioneering work of Dibdin and Reece (5, 6) who developed one- and two-dimensional models to calculate pH profiles in dental biofilms. These early continuous models did not differentiate between the microbial species present and focused only on the diffusive processes associated with the mature dental biofilm (7). They were followed by more advanced examples which aimed to address these issues (8). Arguably, the most complex *in silico* continuous model of the dental biofilm reported so far is the one presented by Ilie et al. (9). In their one-dimensional time-dependent model, the authors included four microbial species (aciduric *Streptococcus*, non-aciduric *Streptococcus*, *Actinomyces*, and *Veillonella*), several metabolic processes (e.g., anaerobic glucose fermentation, aerobic polyglucose conversion, lactate fermentation), Nernst-Plank equations for ion transport, pH calculation and tooth (modeled as hydroxyapatite) demineralization. The microbial growth and the layered structure of the dental biofilm were not considered, with the simplifying assumption that for the simulated time, the microbial composition does not change (9). The model results indicated that *Veillonella* has a protective effect as it consumes the lactic acid produced by aciduric *Streptococcus*. However, recent experimental results contradict this finding and suggest that *Veillonella* may in fact participate in the development of caries through interactions with *S. mutans* (10). Such contradiction between *in silico* and *in vitro* models emphasizes the need for validation and for modeling approaches that allow for the spatial representation of the biofilms (4).

One such approach that is particularly useful for modeling the interactions in polymicrobial biofilm communities and can be appropriately combined with experiments for validation is individual-based modeling (11, 12). Although long established for biofilm research (13), it was not until recently that this type of modeling was used for dental biofilms (14–16). Head et al. (15, 16) modeled a dental biofilm formed by two competing types of bacterial populations, both acidogenic, but only one aciduric and therefore more pathogenic. They focused on microbial function rather than genetic identification of the species and recommended parameterizing biofilm models using experimental data derived from *in vitro* experiments to have meaningful results. Archambault et al. (14) combined individual-based modeling with experimental work in an iterative collaborative process to explore the interactions within a two-species biofilm (*Streptococcus oralis*, an accessory pathogen, and *Lactobacillus paracasei*, an organism with probiotic properties). Although based on a simplified stoichiometry that only takes into account glucose and oxygen, their findings highlighted the value of integrating computational simulations with laboratory experiments for understanding complex oral biofilm dynamics.

In our previous study (17), we experimentally demonstrated the dominance of *S. mutans* in a five-species dental biofilm *in vitro* model, grown in a chemically defined medium (CDM) with different concentrations of substrate (glucose and lactic acid). Here, we have developed mathematical models to simulate the observations of Sangha et al. (17), both for the reactor bulk and for the biofilm grown on hydroxyapatite coupons. The direct connection and comparison between the *in vitro* and *in silico* models are facilitated by the use of a CDM which underpins the defined stoichiometry and kinetics used *in silico*. This represents a significant advancement over prior models by capturing more complex microbial communities than those in previously studied two-species biofilm models. Moreover, our work is among the few *in silico* representations of a complex dental biofilm that has been directly compared with *in vitro* data, making it more practically relevant for understanding biofilm dynamics. This advancement paves the way for assessing the safety of oral biofilm perturbations using a combination of *in vitro* and *in silico* simulations (18).

## RESULTS

In this study, we developed two types of mathematical models to simulate the biofilm reactor experiments reported by Sangha et al. (17), where a continuous flow biofilm reactor was used to mimic the transition toward a cariogenic biofilm (represented by *S. mutans* overgrowth within a five-species dental community). Sangha et al. (17) measured the relative abundance of the five species in both the bulk and biofilm, along with substrate and pH variations over time in the bulk. Our first model is a bulk-phase mass balance, represented as ordinary differential equations (see Supplementary material 1), which we compared to the bulk substrate and pH results from Sangha et al. (17). Our second model is a two-dimensional individual-based model (IbM) of biofilms on hydroxyapatite coupons (see Materials and Methods), which we compared with the relative abundances within the biofilm reported by Sangha et al. (17). Both models were run for varying inlet concentrations of glucose and lactic acid (see Table 1), to replicate the experiments in Sangha et al. (17). The kinetic parameters for the bacterial species (i.e., maximum specific growth rate and substrate affinity constant; see Table 2) were the ones reported in Sangha et al. (17). To study the impact of pH on bacterial abundance in biofilms and *S. mutans* colonization, we ran simulations that accounted for the direct effect of pH on growth rates. We also simulated the effects of various initial seeding strategies on the final bacterial abundance within the IbM. The simulation results are reported together with the corresponding experimental data from Sangha et al. (17), where biofilm samples were collected from the hydroxyapatite coupons on days 2, 3, 5, 7, and 9.

**TABLE 1:** Inlet concentrations in the three experiments reported in Sangha et al. (17)

Experiment description	Glucose concentration (g L^−1^)	Lactic acid concentration (g L^−1^)
Reactor Experiment 1 (RE 1)	21.86	11.61
Reactor Experiment 2 (RE 2)	2.09	12.46
Reactor Experiment 3 (RE 3)	1.98	2.61

**TABLE 2:** Kinetic parameters for the bacterial species considered, Sangha et al. (17)

Bacterial species	Carbon source	μ_max_ (h^−1^)	*K_S_*	
			(mmol·L^−1^)	(g·L^−1^)
*Streptococcus gordonii*	Glucose	0.492	6.6	1.88
*Streptococcus mutans*	Glucose	0.406	5.55	1.00
*Actinomyces oris*	Glucose	0.227	7.78	1.40
*Neisseria subflava*	Glucose	0.261	5.55	1.00
*Veillonella parvula*	Lactic acid	0.246	26.9	2.42

### Representative IbM simulation

**Fig 1:**
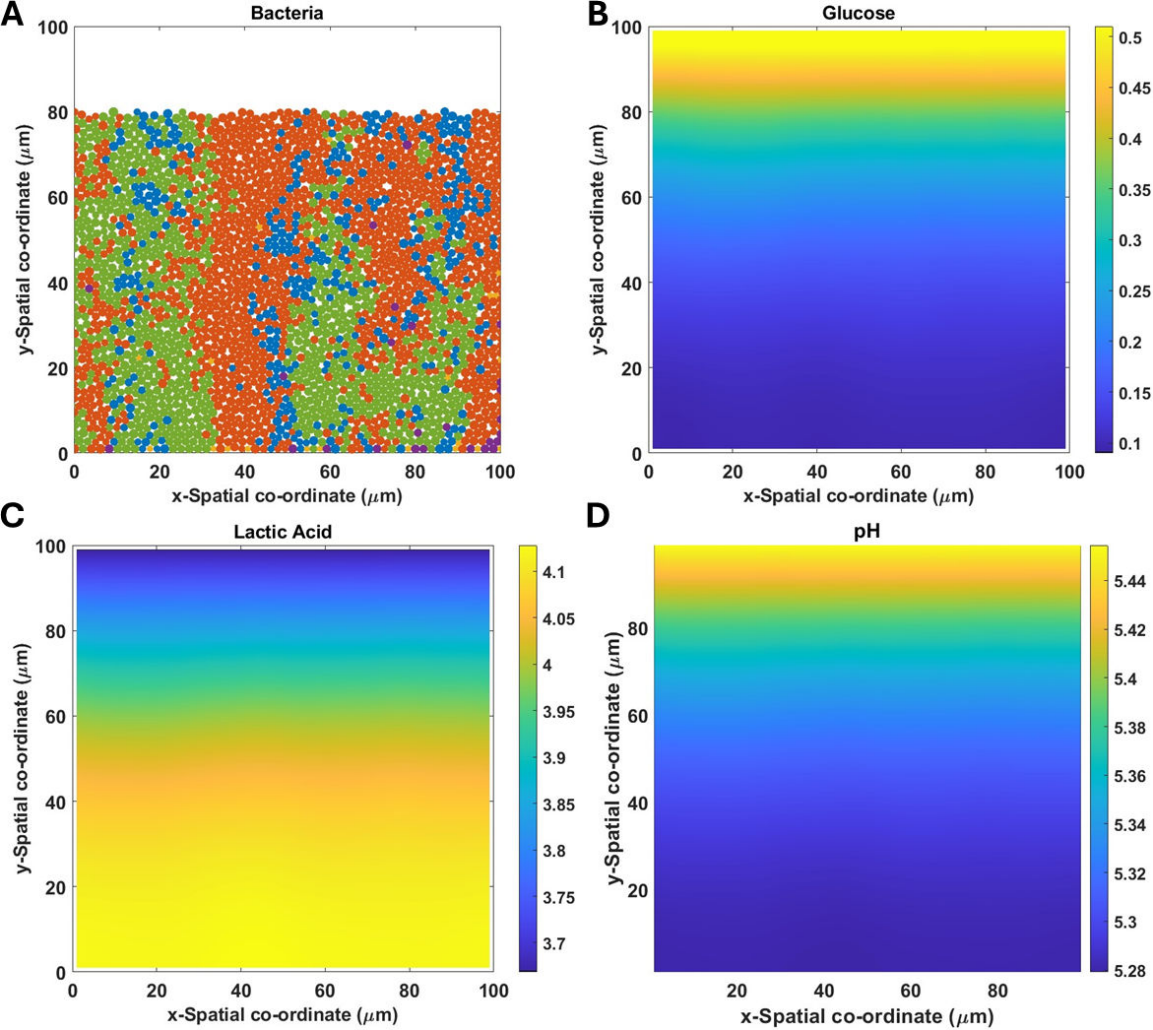
Representation of a biofilm and its corresponding glucose and lactic acid concentration (in g·L^−1^), and pH profiles. The inlet is at the top of the biofilm. The snapshots correspond to RE 3 (low glucose, low lactic acid) at the simulation time of 100 h. (A) Blue*, Streptococcus gordonii*; red*, Streptococcus mutans*; green*, Veillonella parvula;* yellow*, Actinomyces oris*; magenta*, Neisseria subflava*.

The IbM runs were initiated with 46 bacterial cells evenly distributed between the species (nine each from *A. oris, N. subflava, V. parvula,* and *S. mutans,* and 10 from *S. gordonii*) and placed randomly in a monolayer at the base of the computational domain (see further details in Supplementary material 3). Snapshots of the bacterial composition (Fig. 1A) and the corresponding glucose and lactic acid concentrations (Fig. 1B and C, respectively) and pH profile (Fig. 1D) are presented in Fig. 1.

Glucose in the biofilm has the highest concentration at the top and is almost completely depleted at the base (Fig. 1B). The opposite profile is predicted for lactic acid concentration (Fig. 1C). The latter contributes to the pH gradient in the biofilm, which has the lowest value at the base (Fig. 1D). For the dental biofilm height that we have simulated (80 µm), the small variation in the range of pH is similar to the IbM reported by Head et al. (16) and to the homogeneous biofilm in Ilie et al. (9). Similar dental biofilm height was reported in earlier *in vivo* experiments (19). Although the pH range represented is arguably narrow, it shows that there is a spatial variation inside the biofilm (as opposed to the homogeneous liquid phase) and this influences the species’ relative abundance.

### Bacteria relative abundance in the biofilms

**Fig 2:**
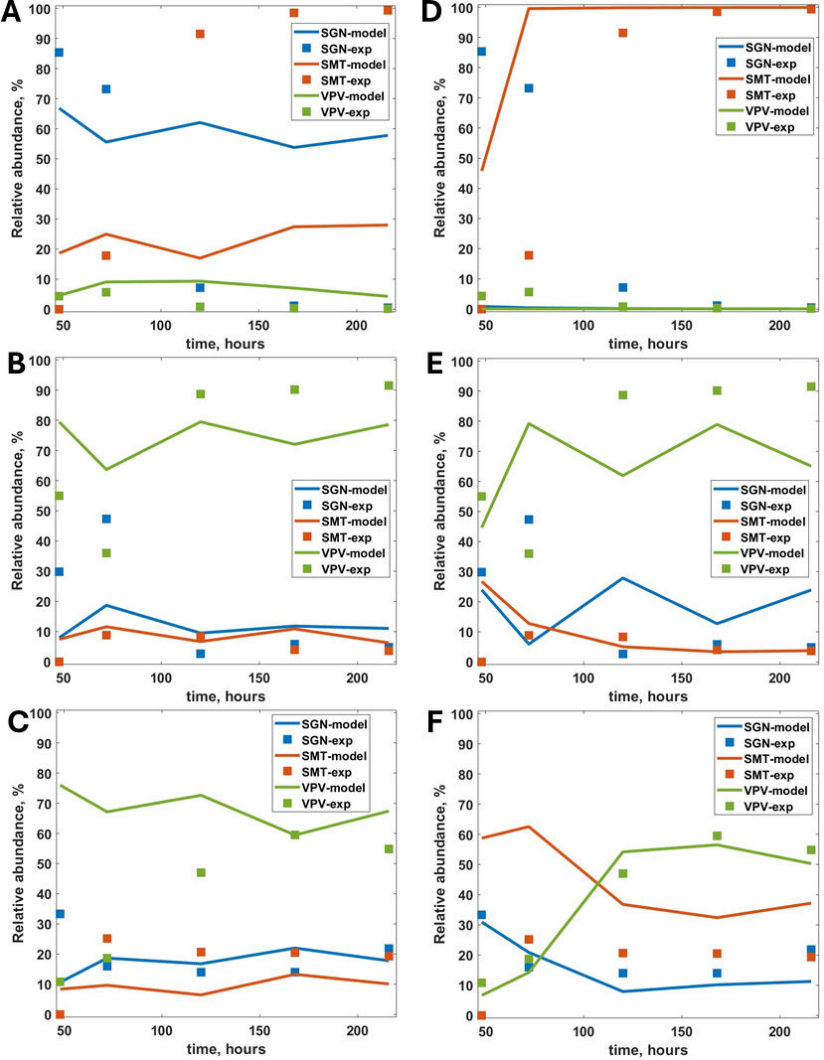
Relative abundance of *S. gordonii* (SGN), *S. mutans* (SMT), and *V. parvula* (VPV) in the biofilm. Model results with and without considering the effect of pH on the growth rate and experimental data for RE 1 (high glucose, high lactic acid; panels A and D), RE 2 (low glucose, high lactic acid; panels B and E), and RE 3 (low glucose, low lactic acid; panels C and F). The simulation results are presented from time = 48 h to correspond with the first experimental point measured. At time = 0, there are 46 bacterial cells evenly distributed between the species (nine each from *A. oris*, *N. subflava*, *V. parvula,* and *S. mutans*, and 10 from *S. gordonii*).

For both the continuous and individual-based models, the bacterial community composition is reported as relative abundance to facilitate the comparison with experimental data in Sangha et al. (17). For the dental biofilm, the first qPCR measurement was taken at 48 h, and therefore the simulation results are represented from this time onward. Without the pH correction for growth rates (Fig. 2A through C), the IbM fails to predict *S. mutans* dominance in the biofilm at high glucose concentrations (Fig. 2A), while *V. parvula* is correctly predicted as the most abundant species at low glucose concentrations regardless of the pH effect (Fig. 2B and C). The IbM simulations successfully indicate the dominant bacterial species, both at high and low glucose concentrations, only when the effect of pH on bacterial growth rates was considered (Fig. 2D through F). At high glucose concentrations, *S. mutans* dominates the dental biofilm (Fig. 2D), while *V. parvula* is the species with the highest relative abundance at low glucose concentrations (Fig. 2E and F). The results are similar to the bulk simulations (Supplementary material 1, Fig. S3). In all the simulations, similar to the experimental results reported by Sangha et al. (17), *A. oris* and *N. subflava* levels (no of cells and relative abundance) were very low, and they are omitted from all the following biofilm representation to keep the clarity of the representation.

### Influence of initial bacterial seeding

The IbM simulations reported in the previous section were performed with an initial seeding of the biofilm with an evenly number of individuals from each of the five species considered, randomly distributed at the base of the computational space. We have further studied the influence of the initial cell distribution by (i) clustering the initial individuals by species type and (ii) altering the initial number of individuals. Case (i) was tested as introducing the different species in the reactor on different days could potentially lead to the initial clustering of cells of the same species on the hydroxyapatite coupons. Case (ii) was tested to match the relative abundance measured on the hydroxyapatite coupons on the second day of reactor operation (when *S. mutans* was inoculated) as reported in Sangha et al. (17) and included in Table S4. For case (ii), we have run three scenarios, considering *S. mutans* seeding with one, three, and five individuals (out of a total of 46 cells initially seeded), respectively, the other species being scaled to maintain relative abundance. All the following simulations were performed considering the effect of pH on the growth rates.

**Fig 3:**
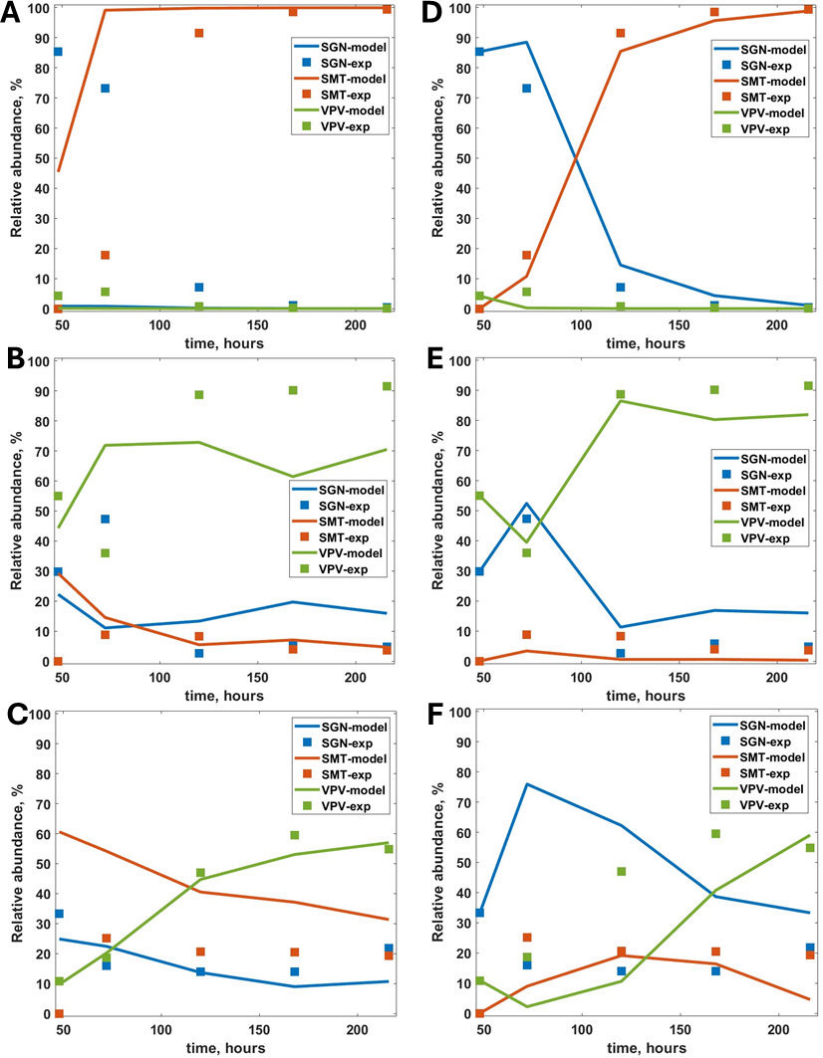
Relative abundance of *S. gordonii* (SGN), *S. mutans* (SMT), and *V. parvula* (VPV) in the biofilm for different initial seeding. Model results and experimental data for RE 1 (high glucose, high lactic acid; panels A and D), RE 2 (low glucose, high lactic acid; panels B and E), and RE 3 (low glucose, low lactic acid; panels C and F). The simulation results are presented from time = 48 h to correspond with the first experimental point measured. At time = 0, in panels A–C**,** there are 46 bacterial cells (nine each from *A. oris, N. subflava, V. parvula,* and *S. mutans,* and 10 from *S. gordonii*). For panels D–F, at time = 0, there is only one individual of *S. mutans*, while the other four species have the relative abundance measured on day 2 of the experiments reported in Sangha et al. (17) and included in Table S4.

Changing the initial arrangement of the seeding cells from randomly distributed to spatially clustered by species type (case (i)) did not significantly change the final relative abundance in the biofilm, with *S. mutans* still dominating at high glucose, and *V. parvula* at low glucose concentrations (Fig. 3A through C). For case (ii), even when starting with only one *S*. *mutans* cell, this species successfully establishes and dominates the biofilm at high glucose concentrations, while *V. parvula* is the most abundant at low glucose and lactic acid concentrations (Fig. 3D through F). Similar results were obtained when *S. mutans* population was initiated with three and five individuals, though for the low glucose, low lactic acid case (RE 3), *V. parvula* relative abundance is significantly lower (Fig. S5, Supplementary material 4).

## DISCUSSION

Our models were successful in predicting the dominant species in the community after 9 days, but only when a pH correction for growth rates was included in the growth rates of bacteria.

In the absence of a more detailed representation of the metabolic processes beyond the overall catabolic and anabolic reactions, the pH correction for growth rates can signify the acid tolerance response/adaptation in bacteria (20). Our results emphasize therefore the importance of considering the pH effect not only on the speciation of the main chemical species included in the stoichiometry of the biological transformations (21) but also on the maximum growth rates of bacteria. Nevertheless, this renders the simulation results very sensitive to the parameters considered for each species in equation 5 (see Table 4) and directly influences the bacterial capacity to establish in the biofilm. For example, we have considered a lower *pH*_*min*_ and *pH*_*opt*_ for *S. mutans* (4 and 6 respectively) than the other four species in the model (Table 4). This is aligned with previous modeling papers (15, 16) and supported by the fact that this species can continue to grow in continuous cultures at pH values of 4.5–5.0 (22). *S. mutans* is also known to continue to perform glycolysis and membrane proton transport at pH 2.5–3.0 (23). Moreover, in studies with dental biofilms, the local pH falls regularly below 4.0 (24) and *S. mutans* biofilm survived acid stress of pH 3.0 (25). Using these pH reference values, *S. mutans* successfully establishes in the biofilm. However, changes in the pH values can significantly alter simulation results. For instance, in one simulation (data not shown), when considering *pH*_*opt*_ for *S. mutans* equal to 7 (rather than 6) and starting from the relative abundance measured on day 2 in the experiments reported in Sangha et al. (17), *S. mutans* did not establish in the biofilm. Moreover, while we have used literature-derived values to implement the pH correction for growth, obtaining the corresponding parameters experimentally for the exact species under study will likely give more realistic results.

Our explicit way of considering the pH effect for each species’ growth rate allows a more flexible representation of the biofilm development and better reflects the influence of the local pH variation on the biofilm growth. Nevertheless, it also links and limits the predictive capacity of the models to the quality of the experimental data they are based on. The way we have considered pH influence differentiates our model from the one proposed by Head et al. (16, 26) who have integrated the pH influence as an inhibition term. Other recent cellular automata models for dental biofilm do not include the pH influence or chemical speciation (27, 28). In the continuous model of Ilie et al. (9), there is no microbial growth and therefore, although their model considers more chemical processes, it cannot be used to simulate the biofilm dynamics.

Our model considers defined stoichiometry for each microbial species, expanding on the previous framework proposed by Gogulancea et al. (21). This allows for studying the changes in microbial composition with time and for the direct comparison with experimental data. This feature is not available in other models, although some authors suggested it as a future development, as species composition influences the potential of caries formation (9). Nevertheless, we have currently not modeled the transition between aerobic and anaerobic metabolism of the species and have included an aerobic bacterium (*N. subflava*) just as a sink for glucose. This is supported by the evidence that supragingival dental biofilms are composed mostly of facultative anaerobes and some aerobes (29), and aerobes are considered to protect the anaerobes in the biofilm (19). Moreover, other *in silico* models of dental biofilms reported that oxygen presence has little effect on acid production and pH, as anaerobic lactic fermentation is the dominant generator of acidity (9). In future development of the model, the transition between aerobic and anaerobic metabolism can be tested by relaxing the assumption of constant oxygen concentration.

The yield variation function of the local substrate concentration surrounding a bacterium differentiates our model from other IbMs of dental biofilms. For example, Head et al. (16, 26) used a constant identical yield for both aciduric and non-aciduric bacterial populations included in their model. Yield calculation based on stoichiometry and local concentrations (equation 1) is important in biofilm modeling as it links environmental conditions to microbial growth (21), thus better reflecting the different conditions bacteria face in a heterogeneous biofilm.

The continuous reactor modeling does not reproduce the dominance of *S. mutans* at low glucose concentrations and the pH profile diverges from experimental data at high pH even after applying pH corrections for the growth rates. These discrepancies may come from the model itself, that is, oversimplifications of the bacterial metabolism, the interactions between bacterial species, or limitations due to what is represented in the model as, for example, not including the contribution of the biofilm forming on the surfaces inside the reactor, noted previously by other authors (7). The first of these could be addressed with further experimental data to quantify the metabolism of each species. Nevertheless, the continuous reactor model shows that all members of the microbial community can co-exist and are the source for biofilm formation and replenishment on the coupons.

A potential use of the dynamic IbM is the simulation of perturbations of the dental biofilm when using personal care products. Using mathematical simulations of biofilm processes was already recognized as a valuable tool in choosing antibacterial approaches (30). Biofilms are up to 100 times more tolerant to antibacterial compounds than the equivalent cells growing planktonically (29). For example, Lee and Kim (31) and Wasfi et al. (32) have shown that the antibacterial activity of *Lactobacillus* species against oral streptococci, and in particular *S. mutans*, is pH-dependent. Our model could be adapted for simulating the integration of other species in the dental biofilm while integrating the effect of pH in the calculations.

One drawback of the 2D model is the limited representation of the mechanical interaction between cells. This is relevant for the dental biofilm as numerous studies have shown that in the presence of sucrose, but not glucose, *S. mutans* forms different colony patterns and invades existing biofilms more effectively. To be able to represent this, the model needs to be translated in 3D as in Gogulancea et al. (21), and to include the production of extracellular polymeric substances as a continuum (33). Such a model would be the most appropriate for studying the effects of antimicrobial agents on the biofilm, for example, to simulate the enzymatic disruption of the biofilm.

## MATERIALS AND METHODS

### Experimental set-up

The experimental setup used to generate the data model in this study is described in Sangha et al. (17). In short, a mixed community formed by five species commonly found in the dental biofilm (*Streptococcus gordonii, Streptococcus mutans, Actinomyces oris, Neisseria subflava,* and *Veillonella parvula*) was grown on hydroxyapatite coupons mimicking the tooth surface in continuous flow bioreactors (Center for Disease Control biofilm reactor) operated for 9 days, at a flow rate of 0.4 mL min^−1^ and fed with a CDM with different concentrations of glucose and lactic acid. The inoculation procedure was as follows: on day 0, *A. oris*; on day 1, *S gordonii*, *N. subflava* and *V. parvula*; and on day 2, *S mutans*. The concentrations of glucose and lactic acid in the reactor bulk were measured every day, and the pH was recorded continuously. The relative abundance of the bacterial species in the bulk and on the hydroxyapatite coupons was estimated by qPCR (17).

The kinetic parameters (maximum specific growth rate μ_max_ and substrate affinity constant *K*_*S*_) are unique for each species and were measured experimentally assuming Monod kinetics with glucose and lactic acid as limiting carbon sources as relevant (Table 2).

### Mathematical model description

**Fig 4:**
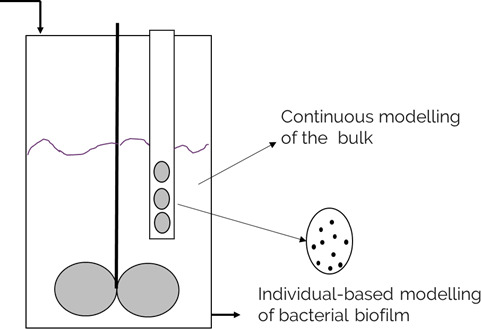
Schematic representation of the experimental setup and the model used for each of the reactor sections. For modeling the bulk, we assumed that the biofilm contribution to the overall mass balance is negligible. The individual-based model of the dental biofilm is further detailed in Fig. 5.

To describe the phenomena occurring at different scales—in the continuously stirred tank reactor and on the biofilm coupons, two different modeling approaches were used: a continuous 0D mathematical model for the reactor bulk (included in Supplementary material 1) and a 2D IbM for the biofilm formed on the coupons (Fig. 4).

### Bacterial stoichiometry

For each of the bacterial species, an overall growth reaction was proposed (equation 1), based on simplified catabolic and anabolic pathways, as listed in Table 3. S. *gordonii, S. mutans,* and *A. oris* are facultative anaerobes that are assumed to consume glucose (carbon source and electron donor) in anaerobic conditions and produce lactic acid as the main product in dental biofilms (34–36). *V. parvula* is an obligate anaerobe that consumes lactic acid (carbon source and electron donor) producing acetate and propionate (9, 37). *N. subflava* is an aerobic species that consumes glucose and has oxygen as the primary electron acceptor, producing acetate and formate (38). This species was considered as scavenger for oxygen to protect the strict anaerobes, as previously reported for chemostat and biofilm experiments with dental biofilm species (38). We assumed a constant concentration of 1 mg L^−1^ of oxygen, equivalent of the micro-aeration conditions inside the biofilm.

**TABLE 3:** Stoichiometry for the anabolic and catabolic overall reactions for the five bacterial species^*a*^

Chemical species (solutes) and biomass	*S. gordonii* (SGN)		*S. mutans* (SMT)		*A. oris* (ACO)		*N. subflava* (NSB)		*V. parvula* (VPV)	
	Cat	Ana	Cat	Ana	Cat	Ana	Cat	Ana	Cat	Ana
Glu	-1	−0.175	-1	−0.175	-1	−0.175	-1	−0.175	−	−
AcH	−	−	−	−	−	−	2	−	0.333	−
LacH	2	−	2	−	2	−	−	−	-1	−0.35
ForH	−	−	−	−	−	−	1	−	−	−
PropH	−	−	−	−	−	−	−	−	0.667	−
NH_3_	−	−0.2	−	−0.2	−	−0.2	−	−0.2	−	−0.2
O_2_	−	−	−	−	−	−	−1.5	−	−	−
CO_2_	−	0.05	−	0.05	−	0.05	2	0.05	0.333	0.05
H_2_O	−	0.4	−	0.4	−	0.4	−	0.4	−	0.4
H^+^	2	0.05	2	0.05	2	0.05	4	0.05	0.333	0.05
CH_1.8_O_0.5_N_0.2_	−	1	−	1	−	1	−	1	−	1

^*a*^ Glu, glucose; AcH, acetic acid; LacH, lactic acid; ForH, formic acid; PropH, propionic acid.

The overall reaction for the anabolic pathway considers the general biomass formula CH_1.8_O_0.5_N_0.2_ proposed by Roels (39) and uses the methodology described in Heijnen and Kleerebezem (40). The energy required for performing the anabolism is derived from catabolism, during which substrates are converted into lower-energy products, producing ATP.

The stoichiometry of the overall growth reaction is calculated as:


(1) $$OVG=f_{cat}\cdot Cat+Ana$$

where *OVG*, *Ana,* and *Cat* are the stoichiometric coefficients corresponding to the overall growth reaction, anabolic, and catabolic reactions, respectively (Table 3) and *f_cat_* is the defined function of the growth yield as proposed by Heijnen and Kleerebezem (40):


(2) $$f_{cat}=-\frac{1}{Y_{XS}}+\lambda_{ED}$$

where $\lambda_{ED}$ is the stoichiometric coefficient of the electron donor and *Y_XS_* is the biomass yield.

#### Yield estimation

The maximum theoretic growth yield for biomass with respect to the electron donor (*Y_XS_*) was estimated as proposed by Heijnen and Kleerebezem (40). We have chosen this approach (as opposed to a constant yield) as it allows the calculation of the yield function of the local environment (i.e., concentration of reactants and products), a feature particularly important for individual-based model of biofilms. *Y_XS_* (equation 3) accounts for the number of times the catabolic reaction needs to run to generate enough energy for biomass formation and is the ratio between the free Gibbs energy supplied by the catabolic pathway $∆G_{cat}$, and the aggregation of the energy that is required for the anabolic pathway $∆G_{ana}$, and the energy that is dissipated by the bacterial species for maintenance $∆G_{dis}$:


(3) $$Y_{XS}=\frac{\Delta G_{cat}}{\Delta G_{ana}+\Delta G_{dis}}$$

The free Gibbs energy is computed for each catabolic and anabolic reaction using equation 4, with the values for the chemical species-free energy of formation listed in Table S1 in Supplementary material 2.


(4) $$\Delta G_{r}=\Delta G_{r}^{0}+RT\frac{\prod_{i=1}^{n}{\left[Prod\right]}_{i}}{\prod_{j=1}^{m}{\left[React\right]}_{j}}$$

where $∆G_{r}^{0}$ (kJ mol^−1^) represents the free energy of a reaction in standard conditions (1 atm and 20°C); [Prod] (mol L^−1^), the concentration of the reaction products; [React] (mol L^−1^), the concentration of reactants; R (kJ mol^−1^·K^−1^), the ideal gas constant; and T (K) is the temperature. In equation 3, the value used for the dissipation energy is 200 kJ C-mol^−1^, which is in the range reported for aerobic and anaerobic heterotrophs (41).

### pH calculation and correction for the growth rates

The pH variation is modeled explicitly, considering the deprotonation and hydration reactions occurring for the main chemical components of the system (organic acids, CO_2_, and ammonia). The system is buffered using a phosphate buffer in the continuous model, to mimic the experimental system. Due to the computational burden, we employed a carbonate buffer in the IbM, to minimize the number of soluble species and mass balance equations to be solved. The deprotonations are modeled as equilibrium reactions, assuming they occur instantaneously (by comparison with the timescale of bacterial growth and diffusion reactions). The equilibrium reactions and their constants are detailed in Supplementary material 2 (equations S3–S16). The pH is computed at every time step, by solving the corresponding system of mass and charge balances, detailed in Volcke et al (42).

The substrates are considered in one dissociated form, that is, either lactic acid or lactate, not both, assuming bacteria can uptake only one form of a chemical. The concentrations and the availability of these forms are affected by diffusion, mass transfer, and biological processes. The speciation of lactic acid affects *V. parvula* growth, as it can only utilize lactate for growth (9). Glucose, the primary carbon source for the other four species, does not undergo dissociation. The energy balance for the catabolic and anabolic pathways for these species is however influenced by pH which affects the computed values of the growth yields.

To consider the inhibitory effect of pH on the bacterial species growth rate (43), we used the empirical equation proposed by Rosso et al. (44) to model the effect of environmental factors on bacterial growth in predictive microbiology:


(5) $$\mu_{max}\left(pH\right)=\mu_{max,opt}\frac{\left(pH-pH_{min}\right)\left(pH-pH_{max}\right)}{\left(pH-pH_{min}\right)\left(pH-pH_{max}\right)-{\left(pH-pH_{opt}\right)}^{2}}$$

The parameters *pH_min_, pH_opt_,* and *pH_max_* are specific to each bacterial species, as species have different tolerances to acidic and alkaline conditions and were derived from the literature (Table 4).

**TABLE 4:** Parameters of equation (5) for each of the species in the model

Species	${pH}_{min}$	${pH}_{opt}$	${pH}_{max}$	Reference
*S. gordonii*	4.5	7	9	(45)
*S. mutans*	4	6	9	(16, 45, 46)
*A. oris*	5.3	7	9	Proposed based on reference (47)
*N. subflava*	5	7	9	Proposed based on reference (48)
*V. parvula*	4.8	7	9	Proposed based on reference (15)

### Individual-based model

In the IbM, the bacterial cells are represented as discrete entities (particulate components of the model), while the chemical species involved in the biochemical reactions (see Table 3) are soluble components (solutes) that participate in diffusion-reaction processes, generating the field of concentration.

**Fig 5:**
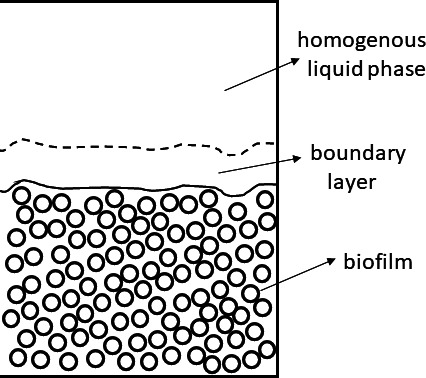
Schematic representation of the subdomains included in the IbM. In the homogeneous liquid phase and boundary layer subdomains, there are only soluble components (i.e., glucose, lactic acid, acetate, etc) and no biomass, while in the biofilm subdomain, there are both biomass (bacterial cell) and soluble components.

The two-dimensional biofilm model is split into three subdomains (Fig. 5): the biofilm itself, growing at the bottom of the domain; the boundary layer; and the homogeneous liquid phase.

Bacterial cells are modeled as cylinders of constant height (1 µm), each bacterial agent having its own set of properties (radius, mass, set of kinetic parameters). The bacterial cells have a maximum (division) radius of 2 µm (Head et al. (16)). The initial seeding radius for all the cells was set at 90% of the division radius. Each bacterial cell has its unique sets of spatial coordinates, specifying the position of its center and its radius. The cells are placed in a 2D computational domain, a square of 100 × 100 µm, considered representative of the biofilms formed on the hydroxyapatite coupons. The bacterial biofilm is allowed to reach the maximum height of 80 µm (16). Bacterial cells above this height are removed from the computational domain.

The bacterial growth and substrate consumption were modeled with a mixed thermodynamic—empirical Monod approach, previously described in Gogulancea et al. (21): the growth kinetics are described by a traditional Monod calculation and the bacterial yield is estimated using a thermodynamic approach based on catabolism and anabolism stoichiometric equations of each species (equation 3). The IbM allows for three possible behaviors of bacterial cells:

- *Growth*: If the growth requirement ($\mu_{max}\frac{S}{K_{S}+S}$) is higher than its maintenance requirement (*m*), the cell is allowed to grow, using the following mass balance equation
(6) $$\frac{dX_{j}\left(x,y\right)}{dt}=\left(\mu_{max}\frac{S\left(x,y\right)}{K_{S}+S\left(x,y\right)}-m\right)X_{j}\left(x,y\right)$$
  where *µ_max_* (h^−1^) is the maximum specific growth rate; Ks (mmol L^−1^) is the substrate affinity constant; *S(x,y*) is the limiting substrate concentration in the grid cell (x,y)—either glucose or lactic acid, depending on bacterial species; *m* maintenance term calculated with equation 7; and $X_{j}\left(x,y\right)$ the concentration of biomass of species *j* in the grid cell (x,y).
- *Maintenance*: The maintenance term considers the Gibbs-free energy of dissociation and catabolism, defined by equation 7:
(7) $$m=\frac{m_{G}}{\Delta G_{cat}}$$
  where *m_G_* is the maintenance energy, considered constant for all the species and equal to 4.5 kJ C-mol^−1^ h^−1^ (40). If the growth requirement is equal to its maintenance requirement, the cell maintains its current mass:
(8) $$\frac{dX_{j}\left(x,y\right)}{dt}=0$$
- *Decay*: if the growth requirement is higher than the maintenance requirement, the cell enters the decay stage
(9) $$\frac{dX_{j}\left(x,y\right)}{dt}=-k_{d}X_{j}\left(x,y\right)$$

where $k_{d}$ is the decay coefficient, assumed to have the value of 4.2·10^−3^ h^−1^, similar to Head et al. (16).

As bacteria grow, they reach the maximum imposed radius and then divide into two individual cells, each containing between 45% and 55% of the initial parent cell mass. One of the daughter cells retains the position of the parent, while the other is placed adjacent to it, at a random angle. After each successful integration step, a division check is performed, and the new numbers and positions of the bacterial cells are recorded. As cells might overlap following a division event, an overlap check is performed and overlaps are resolved by pushing the existing neighbor cells until the maximum overlap between bacterial cells becomes lower than 10% (of their area). This process is traditionally called “shoving” and it was initially described in Kreft et al. (13). During the decay stage, the cell shrinks, and the biomass is transformed back into soluble components (the reverse of the anabolic reaction). When the radius of a bacterial cell becomes smaller than 10% of its radius following division, the cell is removed from the biofilm.

#### Solute mass balance

In the biofilm subdomain, it is assumed that the solutes are transported only by diffusion, which is modeled using Fick’s second law. The mass balance equation for the solute *i* (see Table 3) in the grid cell of the coordinates (*x,y*) is therefore:


(10) $$\frac{\partial S_{i}}{\partial t}=D_{eff}\left(\frac{\partial^{2}S_{i}}{\partial x^{2}}+\frac{\partial^{2}S_{i}}{\partial y^{2}}\right)+\sum r_{ij}$$

where *S_i_* is the molar concentration of chemical species *i*, *D_eff_* is the effective diffusion coefficient of chemical species *i*, and $\sum r_{ij}$ is the net reaction term for the chemical species *i*. The net reaction term represents the sum of the rates of all the processes in which the soluble component *i* is involved (see Table 3), weighted by the yield factors corresponding to the particular bacterial cell type *j* (equation S1 in Supplementary material). The effective diffusion coefficient considers that in the biofilm subdomain, the diffusion of solutes is affected by the presence of cells that have higher density than the liquid. Therefore, in the biofilm subdomain, the diffusion coefficients in water are adjusted with a diffusion factor $d_{f}\left(x,y\right)$ proposed by Fan et al. (49) and further used in the IbM proposed by Ofiteru et al. (50):


(11) $$d_{f}\left(x,y\right)=1-\frac{0.43\cdot X{\left(x,y\right)}^{0.92}}{11.19+0.27\cdot X{\left(x,y\right)}^{0.99}}$$

where $X\left(x,y\right)$ represents the total biomass concentration in the grid cell with the coordinates $\left(x,y\right)$.

#### Boundary layer

On top of the biofilm, there is a 20 µm boundary layer, in which we assume the entire diffusional resistance of the liquid is concentrated. No chemical reaction occurs in the boundary layer, and the diffusion coefficients for the soluble species are the same as those reported for water (values presented in Supplementary material 2, Table S2).

Therefore, the mass balance for the soluble components in the homogenous liquid phase and the boundary layer is also described by equation 10 but without the reaction term. In the boundary layer, the diffusion factors $d_{f}\left(x,y\right)$ are equal to 1 (as *X(x,y*) =0).

In the bulk subdomain, the diffusion coefficients are assumed to be several orders of magnitude higher than in the boundary layer to describe the perfectly mixed environment on the top of the biofilm, which has the same concentrations as the bulk liquid in the reactor (50).

The model assumes that the biofilm exists within a continuous reactor setup, whose behavior is solely influenced by the biofilm, with no suspended microbial growth occurring in the reactor, as presented originally in Picioreanu et al. (51).

The mass balance equations for the reactor are as follows:


(12) $$\frac{dS}{dt}=\frac{\sum_{i=1}^{n}r_{liq,i}}{V_{reactor}}+\frac{S_{0}-S}{\tau }$$

where $\sum_{i=1}^{n}r_{liq,i}$ is the sum of all the reaction rates at which the soluble species are consumed or produced by each bacterial agent *i* located in the biofilm subdomain. We assume the biofilm volume represents a small fraction of the reactor volume (its characteristic length is assumed to be 100 times larger than a biofilm grid cell).

### Simulations

The model was implemented in Matlab and the general solving algorithm is presented in Fig. S4 in Supplementary material 3. The model was run for a total simulation time of 9 days. The IbM simulations were initially run in triplicates, to verify the random division positioning and overlap resolve do not significantly impact the outcomes of the model. The average simulation time on a computer with 11th generation Intel Core i7 @2.30 GHz processor and 16 GB RAM was 48 h.

## Data Availability

Data created during this research are available at Newcastle University Research Data Archive at https://doi.org/10.25405/data.ncl.c.7076210.v1

## SUPPLEMENTAL MATERIAL

Supplemental material msphere.00743-24-s0001.docx Supplemental text, figures, and tables.

ASM does not own the copyrights to Supplemental Material that may be linked to, or accessed through, an article. The authors have granted ASM a non-exclusive, world-wide license to publish the Supplemental Material files. Please contact the corresponding author directly for reuse.
