# Supplementary material for: Advancing dental biofilm models: the integral role of pH in predicting S. mutans colonization
Source: mSphere. 2024 Dec 11;10(1):e00743-24. doi: 10.1128/msphere.00743-24 (PMC11774048; doi:10.1128/msphere.00743-24)
Supplement: Supplemental material — Supplemental text, figures, and tables. [file msphere.00743-24-s0001.docx]

**Supplementary material 1**

**Continuous stirred tank reactor**

**Model description**

The synthetic community growth in the suspended culture of the CDC reactors reported in Sangha et al (2024) was modelled as a 0-D continuous model. The continuous model allows for the simulation of a homogenous mixed species system, and the comparison of the relative abundances in the liquid culture against those measured in the colonisation experiments reported in Sangha et al (2024), whilst predicting substrate concentration and consumption, and pH in the bulk. The model assumes that the bulk is a homogenous mixture, at constant temperature.

The continuous model is comprised of 15 ordinary differential equations which represent the mass balances for the 10 chemical species (eq. S1) included in the stoichiometry (see Table 3) and the 5 bacterial species (eq. S2).

$\frac{dS_{i}}{dt}={\sum_{j} \frac{1}{Y_{i,X_{j}S}}\mu}_{j}X_{j}+\frac{1}{\tau}\left( S_{i,0}-S_{i} \right)$ (S1)

where *S_i_* is the concentration of solute *i* (mol L^-1^), S*_i,0_* is the inlet concentration to the bioreactor (mol L^-1^), $Y_{i,X_{j}S}$ is the yield for the *j*-bacterial species growing on/producing the chemical species *i* (mol-X mol-S^-1^), $\mu_{j}$ and $X_{j}$ are the corresponding growth rate (h^-1^) and concentration for bacterial species *j (*mol L^-1^*),* respectively and *τ* is the reactor residence time (h^-1^). The growth yields are calculated at every time step (eq. 3 in the manuscript), to account for the changing conditions in the reactor.

$\frac{dX_{j}}{dt}=\mu_{j}X_{j}+\frac{1}{\tau}\left( X_{j,0}-X_{j} \right)$ (S2)

where *X_j_* is the concentration of bacterial species *j* (mol L^-1^), $X_{j,0}$ is the inlet concentration of the bacterial species *j* (mol L^-1^; set to zero for all the species), $\mu_{j}$ is the growth rate (h^-1^) of the species *j* and *τ* is the reactor residence time (h^-1^). No explicit decay is considered in the bioreactor model, under the assumptions that in the continuous reactor model the dead cells are accounted for by the outlet flow.

For the continuous model, the synthetic community members were introduced in the simulation at the same times as in the inoculation procedure reported in Sangha et al (2024): on day 0, *A. oris*; on day 1, *S gordonii*, *N. subflava* and *V. parvula*; and on day 2, *S mutans*. The model was implemented in Matlab® and integrated with Runge Kutta numerical method (ode15s function). The simulation results give all the soluble species’ concentration and the biomass, and computes and updates the pH values after every successful integration step. The results are plotted against data from the *in vitro* experiments. For the bacterial species, the final results are calculated as relative abundances, in order to be able to compare them with the data collected from the *in vitro* system, while for the substrates concentration and pH there is a direct comparison with the measured data.

***Initial conditions***

The continuous model simulations followed the experimental seeding strategy to allow a bacterial community to form before the invasion by *S. mutans* Sangha et al (2024). Thus, in the first 24 h of simulation the only species present was *A. oris*, after which *S. gordonii, N. subflava* and *V.parvula* were added, followed by *S. mutans* at 48 h. The initial biomass concentration for each species was 1.47e-4 mmol L^-1^ while the substrates concentration (initial and in the feed) and the feed flowrate were the same as in the experiments reported in Sangha et al (2024).

***Predicted and measured glucose and lactic acid concentration***

The model simulations for substrates concentration are presented in Figure S1. There are significant discrepancies in the variation patterns compared to the experimental data, especially for the lactic acid simulations, and in particular at high concentrations (Figure S1 A and D). This may be due to the simplified stoichiometry considered for the bacterial species which consider only a very limiting number of substrates (glucose and lactic acid) while they grow in a complex medium.

| **Glucose** | **Lactic acid** |
| --- | --- |
| **A**  **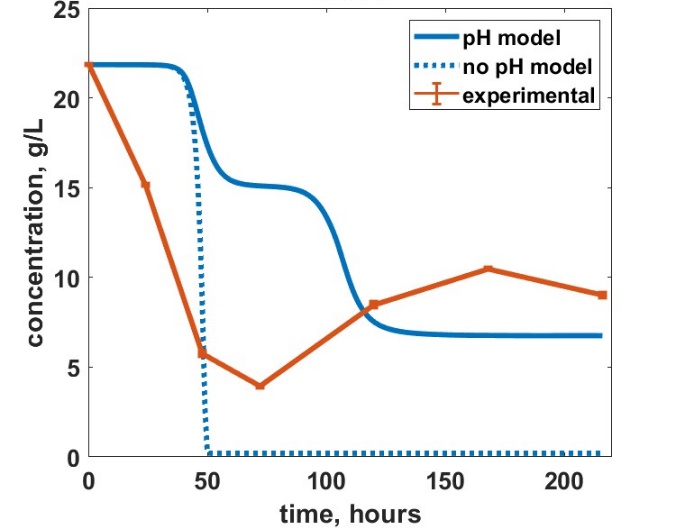** | **D**  **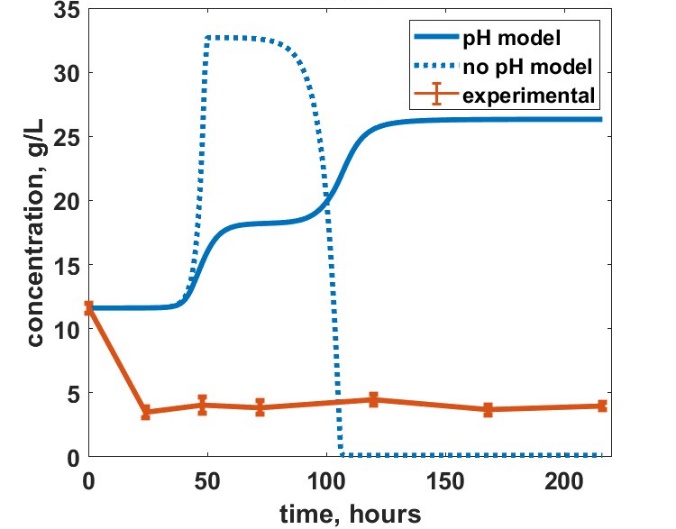** |
| **B**  **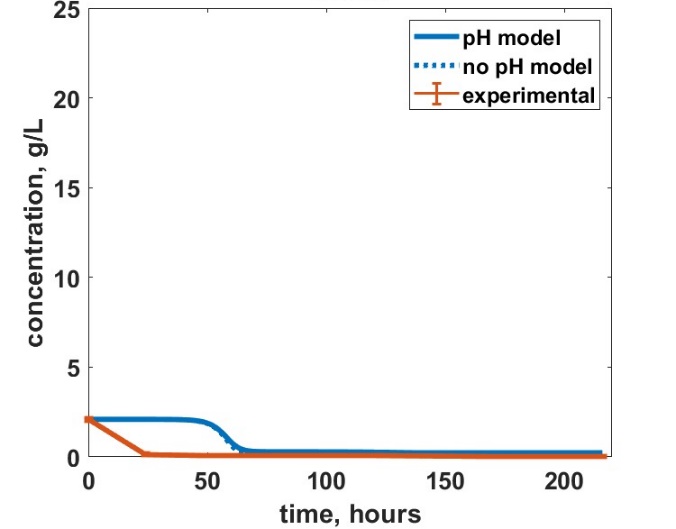** | **E**  **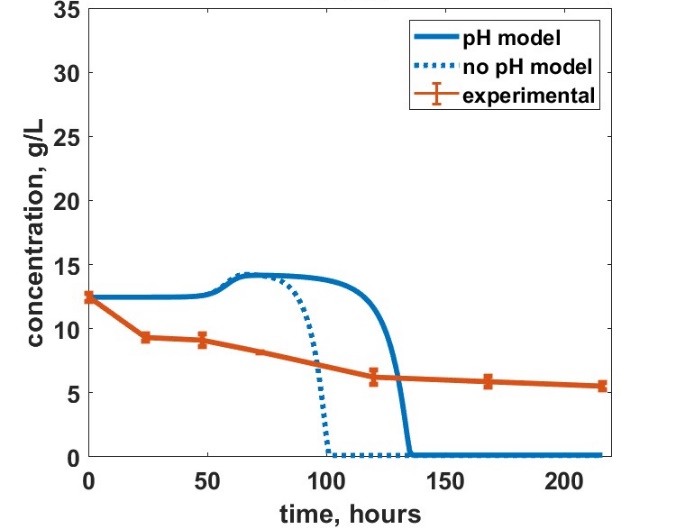** |
| **C**  **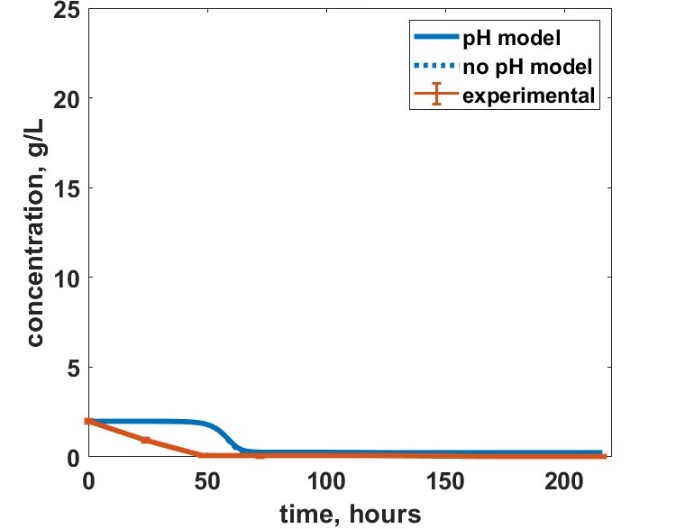** | **F**  **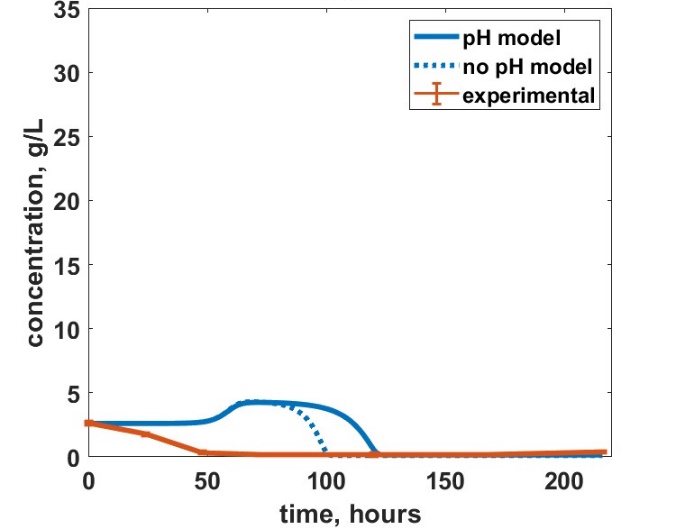** |

**Figure S1. Glucose (left column panels) and lactic acid (right column panels) concentrations**, model with and without pH influence on the growth rates and experimental data for RE 1 (high glucose, high lactic acid; panels A and D), RE 2 (low glucose, high lactic acid; panels B and E), and RE 3 (low glucose, low lactic acid; panels C and F).

***Predicted and measured pH profiles in the bulk***

In all the simulations there is a significant decrease in pH after 48 h, when all the five species are added in the system (Figure S2). Although this decrease comes later than in the experimental data, simulations of the pH in the bulk follow the experimental pattern. The predicted steady-state level (at 9 days) is however lower than in the experiments with high lactic acid levels (Figure S2 A-B), and the system is only quantitively matching at low glucose and lactic acid concentrations (Figure S2, C). The pH decrease prediction in RE 1 mirrors the lactic acid production.

| **A**  **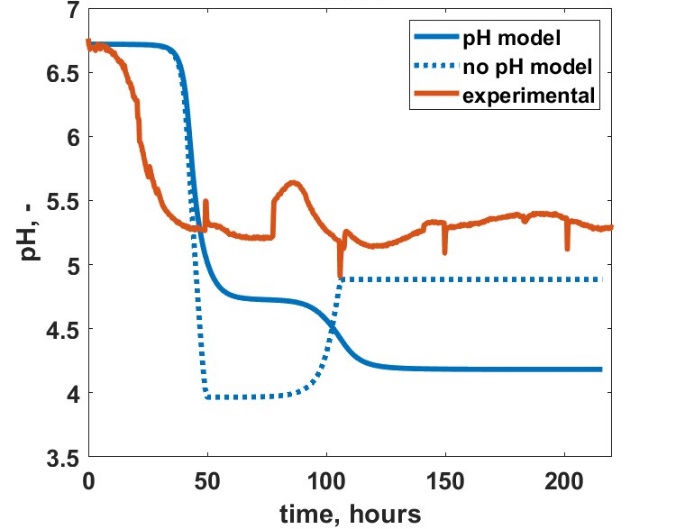** | **B**  **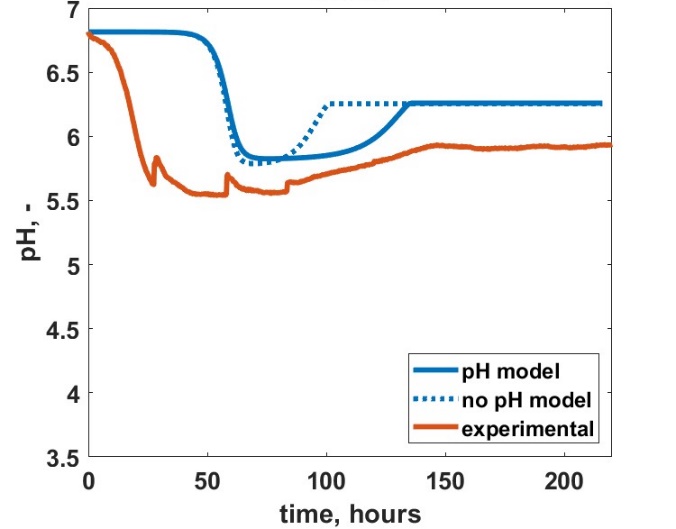** |
| --- | --- |
| **C**  **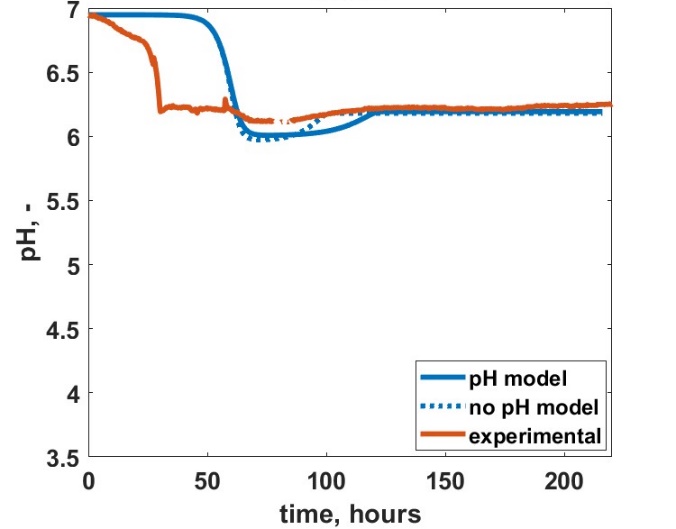** | |

**Figure S2. pH profile in the CDC reactor bulk**, model with and without pH influence on growth rates and experimental data for RE 1 (high glucose, high lactic acid; panel A), RE 2 (low glucose, high lactic acid; panel B) and RE 3 (low glucose, low lactic acid; panel C).

***Predicted and experimental bacterial relative abundance***

The bacterial community composition is reported as relative abundance to facilitate the comparison with experimental data. The continuous model simulations which do not include the pH correction for growth rates predict the domination of *V. parvula* in the bulk irrespective of the inlet concentrations of glucose and lactic acid (Figure S3, A – C). *V. parvula* is the only species which consumes lactic acid, which is both fed in the reactor and produced by the metabolism of other three species (*S. gordonii, S. mutans* and *A. oris*). Therefore, *V. parvula* does not face substrate limitation and dominates the bulk, with relative abundance more than 85% in the simulations with high glucose and high lactic acid (RE 1; Figure S3 A). In the simulations with low glucose concentrations, *V. parvula* growth is accelerated, making up 98% of the bulk (RE 2 and RE 3, Figure S3 B - C).

| **Without pH correction for growth** | **With pH correction for growth** |
| --- | --- |
| **A**  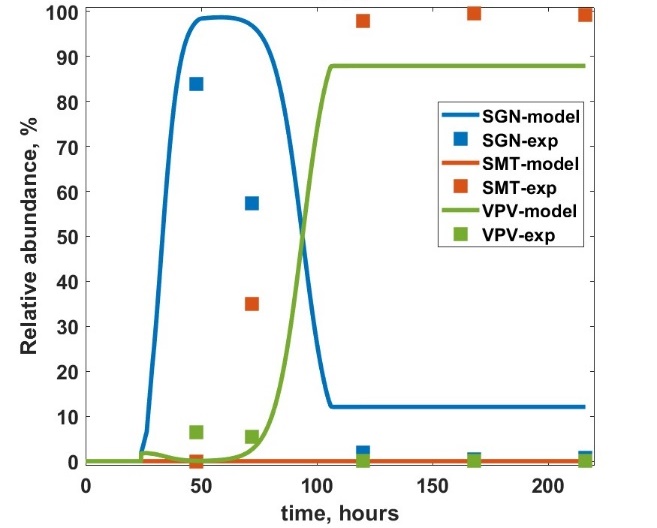 | 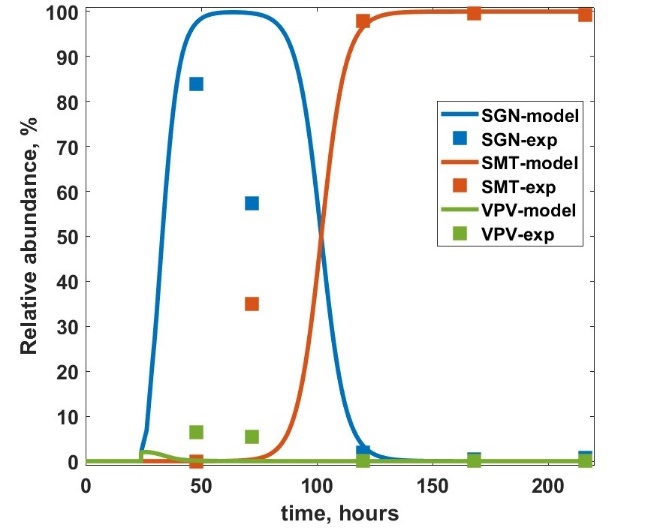**D** |
| 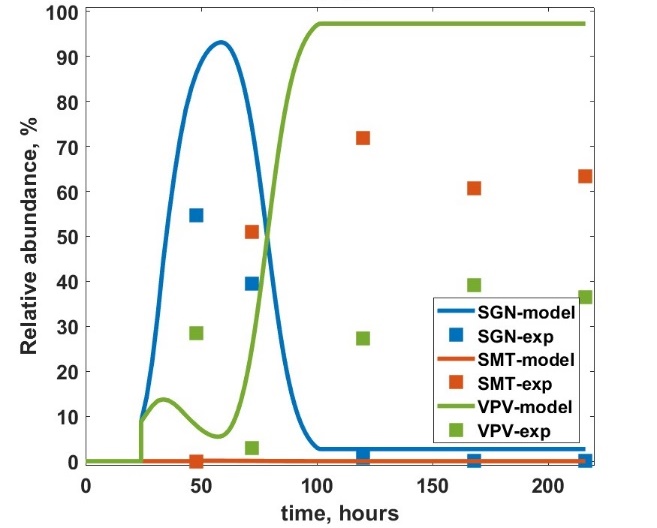**B** | 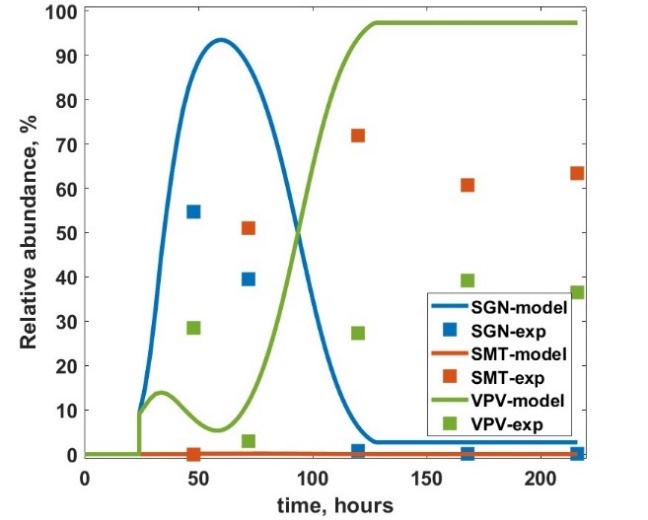**E** |
| **C**  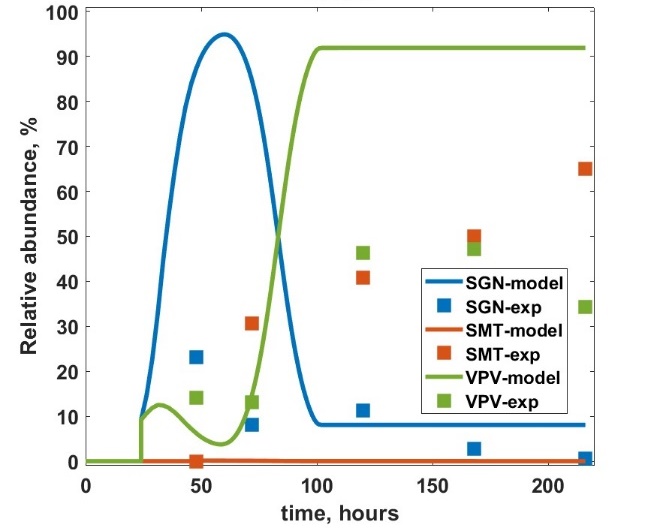 | **F**  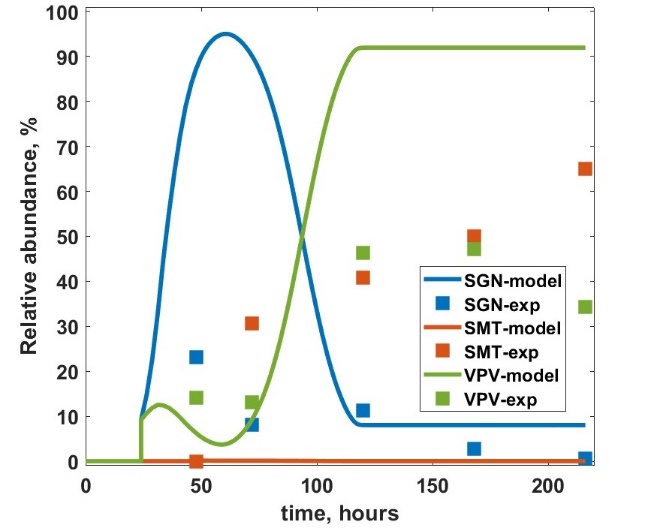 |

**Figure S3. Relative abundance of *S. gordonii* (SGN), *S. mutans* (SMT) and *V. parvula* (VPV) in the continuous reactor**. Model results without pH (A-C) and with pH (D-F) and experimental data for RE 1 (high glucose, high lactic acid; panels A and D), RE 2 (low glucose, high lactic acid; panels B and E), and RE 3 (low glucose, low lactic acid; panels C and F).

This is in contrast with the experimental results, which indicates dominance of *S. mutans* in all three conditions studied. In the case of high inlet glucose concentrations, experimental data indicated that *S. mutans* dominates the bulk soon after its addition at 48 h, and by day 5 represent 99% of the community. The continuous model does not reflect this when the pH correction for growth rates is not included, in which case *S. gordonii* is the dominant *Streptococcus* species as it has the higher maximum specific growth rate. After the initial growth of *S. gordonii*, as glucose is consumed and lactic acid is produced, *V. parvulla* dominates the bacterial population in the reactor. Steady state is reached after 110 h for the case of the higher inlet glucose concentration and 100 hours for the case of the lower inlet glucose concentration. For the lower inlet glucose concentration, glucose is consumed faster and the relative abundance of *S. gordonii* drops to 4%. The inlet glucose concentration is shown to have the most important impact: experimentally, at lower inlet concentrations, *S. mutans* is still successful in establishing in the community but its relative abundance only reaches 62% after nine days, while the *V. parvula* accounts for the remainder 38% of the planktonic bacteria. The continuous model, without taking into account pH correction for growth rates, is only able to qualitatively describe the behaviour of *V. parvula* and *S.gordonii*, but not the invasion of *S. mutans* at low glucose concentration.

When pH correction for growth rates is included, the simulations indicate that *S. mutans* establishes and dominates the bulk at high glucose concentration (Figure S3, E – F). This is due to the pH value decreasing to under 4.5, when only *S. mutans* can grow based on the pH ranges proposed in Table 3. However, at low glucose and lactic acid concentrations, when the pH in the bulk stabilizes at higher values (see Figure 2, C-D), the continuous model with pH correction for growth rates fails to reproduce *S. mutans* dominance in the bulk, in contrast to the experimental results (Figure S3, G – H).

**Supplementary material 2**

**Thermodynamic properties**

***Table S1* Gibbs free energy of formation** **for chemical compounds**

| ***Component*** | ΔG^0^_f_ (kJ/mol) | Reference |
| --- | --- | --- |
| ***Glucose*** | -915.9 | *(*Heijnen and Kleerebezem 2010*)*  Perry and Green (2008) |
| ***Acetic acid***  (CH_3_COOH) | ΔG_f,_ _CH3COOH_ = -396.5  ΔG_f,_ _CH3COO_^-^ = -369.3 |  |
| ***Lactic acid***  (CH₃CHCOOH) | ΔG_f,_ _CH3CHCOOH_ = -430.62  ΔG_f,_ _CH3CHCOO_^-^ = -403 |  |
| ***Formic acid***  (HCOOH) | ΔG_f,_ _HCOOH_ = -493.96  ΔG_f,_ _HCOO_^-^ = -463 |  |
| ***Propionic acid***  (CH₃CH2COOH) | ΔG_f,_ _CH3CH2COOH_ = -291.36  ΔG_f,_ _CH3CH2COO_^-^ = -263 |  |
| ***NH_3_*** | - ΔG_f,_ _NH3_ = -26.57  ΔG_f,_ _NH4+_ = -79.37 |  |
| ***O_2_*** | 16.40 |  |
| ***CO_2_*** | ΔG_f,_ _hydrolysis_ = -386.00  ΔG_f,_ _H2CO3_ = -623.16  ΔG_f,_ _HCO3-_ = -586.85  ΔG_f,_ _CO32-_ = -527.8 |  |
| ***H_2_O*** | -237.18 |  |

***Equilibrium reactions and their constants***

The equations for the pH calculations are detailed below:

LacH ↔Lac^-^+H^+^, $k_{eq}=\frac{\left[ {Lac}^{-} \right]\left[ H^{+} \right]}{\left[ LacH \right]};$ $\left[ {LacH}_{t} \right]=\left[ {Lac}^{-} \right]+\left[ LacH \right]$ (S3)

AcH↔Ac^-^+H^+^, $k_{eq}=\frac{\left[ {Ac}^{-1} \right]\left[ H^{+} \right]}{\left[ AcH \right]};$ $\left[ {AcH}_{t} \right]=\left[ {Ac}^{-} \right]+\left[ AcH \right]$ (S4)

PropH↔Prop^-^+H^+^, $k_{eq}=\frac{\left[ {Prop}^{-1} \right]\left[ H^{+} \right]}{\left[ PropH \right]};$ $\left[ {PropH}_{t} \right]=\left[ {Prop}^{-} \right]+\left[ PropH \right]$ (S5)

FormH↔Form-+H^+^, $k_{eq}=\frac{\left[ {Form}^{-1} \right]\left[ H^{+} \right]}{\left[ FormH \right]};$ $\left[ {FormH}_{t} \right]=\left[ {Form}^{-} \right]+\left[ FormH \right]$ (S6)

NH_3_ + H_2_O ↔NH_4_^+^ + OH^-^ , $k_{eq}=\frac{\left[ {NH}_{4}^{+} \right]\left[ {OH}^{-} \right]}{\left[ {NH}_{3} \right]};$ $\left[ {NH}_{3t} \right]=\left[ {NH}_{4}^{+} \right]+\left[ {NH}_{3} \right]$ (S7)

CO_2_+H_2_O ↔H_2_CO_3_, $k_{eq,1}=\frac{\left[ H_{2}{CO}_{3} \right]}{\left[ {CO}_{2} \right]}$ (S8)

H_2_CO_3_↔HCO_3_^-^ + H^+^, $k_{eq,2}=\frac{\left[ HCO_{3}^{-} \right]\left[ H^{+} \right]}{\left[ H_{2}{CO}_{3} \right]}$ (S9)

HCO_3_^-^↔CO_3_^2-^ + H^+^ , $k_{eq,3}=\frac{\left[ CO_{3}^{2-} \right]\left[ H^{+} \right]}{\left[ HCO_{3}^{-} \right]}$ (S10)

$\left[ {CO}_{2t} \right]=\left[ {CO}_{2} \right]+\left[ H_{2}{CO}_{3} \right]+\left[ HCO_{3}^{-} \right]+\left[ CO_{3}^{2-} \right]$ (S11)

H_3_PO_4_↔H_2_PO_4_^-^+ H^+^, $k_{eq,1}=\frac{\left[ H_{2}PO_{4}^{-} \right]\left[ H^{+} \right]}{\left[ H_{3}{PO}_{4} \right]}$ (S12)

H_2_PO_4_^-^↔HPO_4_^2--^+ H^+^, $k_{eq,2}=\frac{\left[ HPO_{4}^{2-} \right]\left[ H^{+} \right]}{\left[ H_{2}PO_{4}^{-} \right]}$ (S13)

HPO_4_^2--^↔PO_4_^3--^+H^+^, $k_{eq,3}=\frac{\left[ PO_{4}^{3-} \right]\left[ H^{+} \right]}{\left[ HPO_{4}^{2-} \right]}$ (S14)

$\left[ H_{3}{PO}_{4t} \right]=\left[ H_{3}{PO}_{4} \right]+\left[ H_{2}PO_{4}^{-} \right]+\left[ HPO_{4}^{2-} \right]+\left[ PO_{4}^{3-} \right]$ (S15)

H_2_O ↔ OH^-^+ H^+^, $k_{eq}=\left[ H^{+} \right]\left[ {OH}^{-} \right]$ (S16)

The values of the equilibrium constants are retrieved from literature and listed in the Table S2. The concentration of all the species in the system of equations above can be expressed function of the proton [H^+^] concentration and their respective equilibrium constant.

***Table S2.* Dissociation constants**

| Component | Dissociation constant | Reference |
| --- | --- | --- |
| Lactic acid | 10^-3.86^ | (Ilie, van Loosdrecht et al. 2012) |
| Acetic acid | 10^-4.76^ |  |
| Formic acid | 10^-3.75^ |  |
| Propionic acid | 10^-4.86^ |  |
| NH_3_ | 5.6204 · 10^-10^ |  |
| H_2_O | 10^-14^ |  |
| CO_2_ | $k_{eq,1}$ = 1.0081 $k_{eq,2}$  = 5.0 · 10^-7^ $k_{eq,3}$  = 7 · 10^-11^ |  |
| Phosphoric acid | $k_{eq,1}$ = 7.1 · 10^-3^ $k_{eq,2}$  = 6.3 · 10^-8^ $k_{eq,3}$  = 4.2 · 10^-13^ | Washington edu website  [https://depts.washington.edu/eooptic/links/acidstrength.html](https://protect.checkpoint.com/v2/___https://depts.washington.edu/eooptic/links/acidstrength.html___.YzJlOnVsc3RlcnVuaXZlcnNpdHk6YzpvOjMwODM2MDgxMjkxMjI3YzVhYjFlNjg2MTNmYTQyNjMwOjY6ODlhNjpiMTJhNTVjODIyYWE5MjVmODg2MDNjNWUwNWNjMGFiOTY4YmVlNmYzYmUyYTE5YWJmY2U2NmI3ZTgzYzQxNTYzOnA6VDpO) |

The solution is neutral (from the ionic standpoint), and the concentration of all negatively charged species is balanced by that of the positively charged species, as described in the charge balance equation:

$\left[ H^{+} \right]+\left[ {NH}_{4}^{+} \right]=\left[ {Ac}^{-} \right]+\left[ {Prop}^{-} \right]+\left[ {Form}^{-} \right]+\left[ HCO_{3}^{-} \right]+\left[ CO_{3}^{2-} \right]++\left[ {OH}^{-} \right]$ (S17)

The concentrations of all species (function of proton concentration) can be replaced in the charge balance equation (S17) above. The equation is solved using the Newton method for non-linear equations and the pH is expressed using equation (S18) below.

$pH=-\log_{10}\left[ H^{+} \right]$ (S18)

| Soluble component | Diffusion coefficient (m^2^h^-1^) | Reference |
| --- | --- | --- |
| *Glucose* | 3.06 · 10^-6^ | (Ilie, van Loosdrecht et al. 2012) |
| *Acetic acid/Acetate* | 4.97· 10^-6^ |  |
| *Lactic acid/Lactate* | 4.72· 10^-6^ |  |
| *Formic acid/ Formate* | 6.62· 10^-6^ |  |
| *Propionic acid/Propionate* | 4.97· 10^-6^ |  |
| *Ammonia* | 5.90· 10^-6^ | (Yaws 2009) |
| *Oxygen* | 9.58· 10^-6^ |  |
| *Carbon dioxide* | 5.40· 10^-6^ |  |

***Table S3*** Diffusion coefficients for the soluble components considered in the IbM.

**Supplementary material 3**

***Solving algorithm for IbM***

.


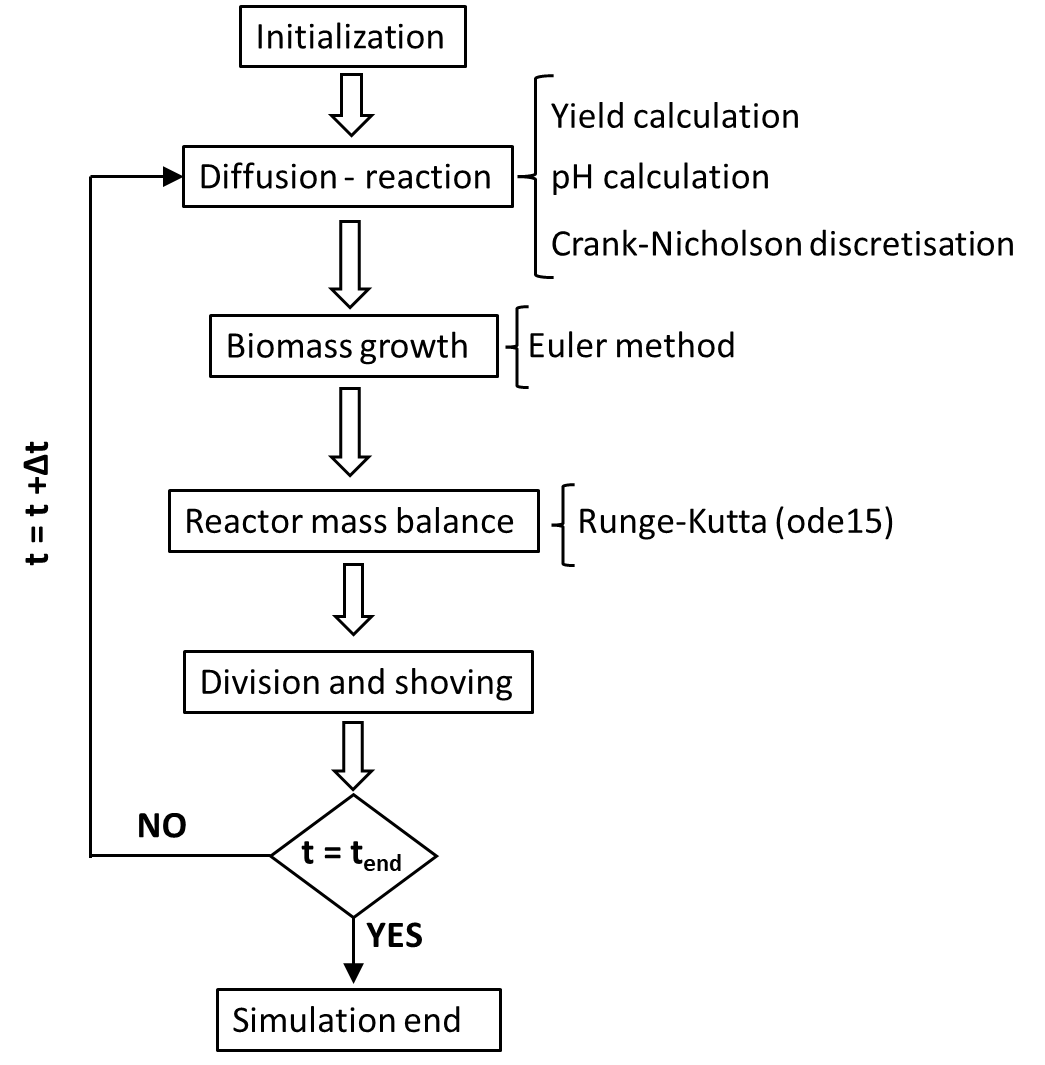


**Figure S4** Solving algorithm for the IbM model

The computational domain (100 x 100 µm) is first seeded with 46 bacterial cells (9 each from *Actinomyces oris, Neisseria subflava, Veillonella parvula* and *Streptococcus mutans,* and 10 from *Streptococcus gordonii*), such that the entire first row of the computational domain is filled with bacterial cells, placed at an equal distance from each other and from the walls of the computational domain. The positions of the agents are randomly assigned.

To solve the reaction-diffusion equations for the soluble species, domain discretization is performed as follows: first, the height of the domain is computed, by adding the height of the boundary layer on top of the height of the biofilm. Then, the computational domain is divided into grid cells, the length and height of the maximum division diameter for the bacterial cells (2 µm). The pH is computed in every grid cell, using the current soluble species concentrations. The reaction rates for each soluble species are computed using the concentration of bacterial agents in each computational grid and the estimated growth yields.

To allow the decoupling of the system of equations for the bacterial and soluble species, we assume that the diffusional processes are much faster compared to the biological processes :the concentration of the bacterial species is considered constant while solving the mass balances for the chemical species (the biological growth takes place on the scale of tens of minutes and the diffusion phenomena has a scale of tens of millisecond) (Kreft, Picioreanu et al. 2001)

The partial differential equation system (PDE) described by equation (10) is transformed into a system of algebraic equations using the Crank-Nicholson discretisation method.

The boundary conditions are as follows:

- Dirichlet boundary condition at the top of the computational domain:

0<x<max_x; y = max_height, S = S0

- The vertical domain walls have periodic boundary conditions

0<y<max_height, x=0, x=max_x, Si=Sj

- No flow condition at the bottom of the computational domain

0<x<max_x; y = 0, dS/dt = 0;

The resulting system is solved iteratively using matrix left division and lower-upper (LU) decomposition. The time step for the diffusion calculations is set at 10^-6^ hours. Following convergence, the soluble species concentrations are updated in every grid cell.

Then, mass balance equations for the bacterial cells are solved: the system of ordinary differential equations (eq. x) is solved using the backward Euler method and a time step of 15 minutes.

The boundary conditions are re-calculated, using the reactor coupling to compute the top boundary conditions for the next time step.

The last steps in the algorithm are the division checks, followed by the overlap resolving. After solving the reactor balance, the mass and radius of each bacterial agent are updated and the division and overlap check is performed.

**Supplementary material 4**

**IbM simulation results for initial seeding with 3 and 5 *S. mutans* cells**

| Random, day 2 distribution, three *S. mutans* cells | Random, day 2 distribution, five *S. mutans* cells |
| --- | --- |
| **A**  **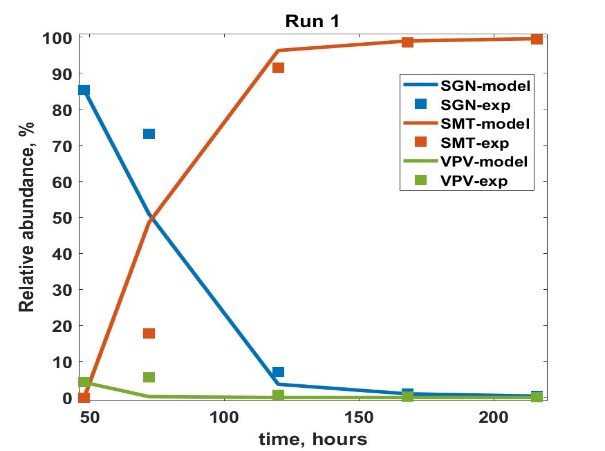** | **D**  **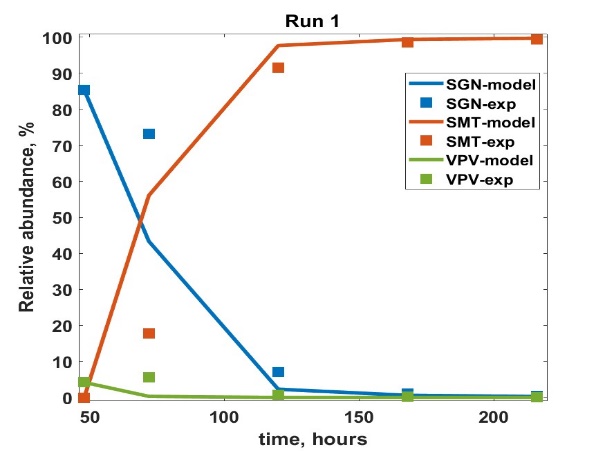** |
| **B**  **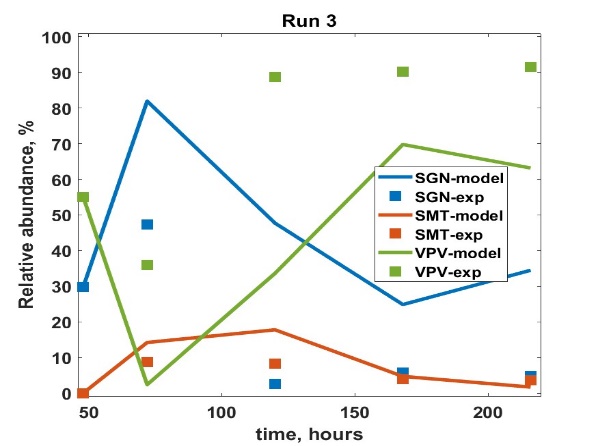** | **E**  **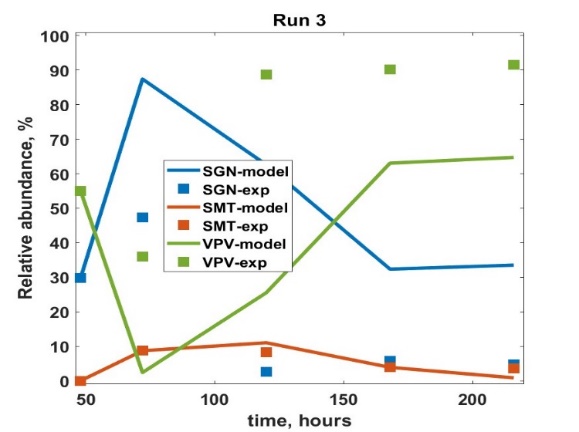** |
| **C**  **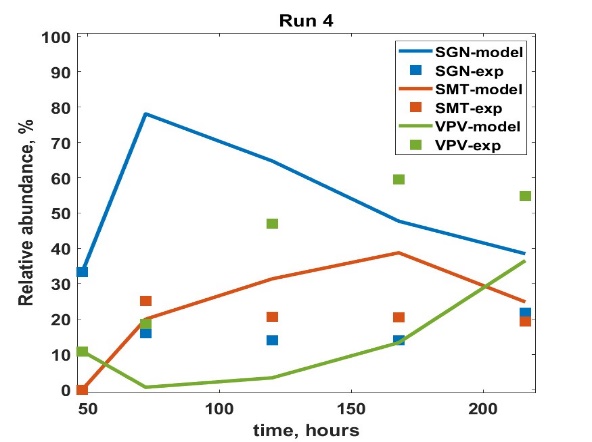** | **F**  **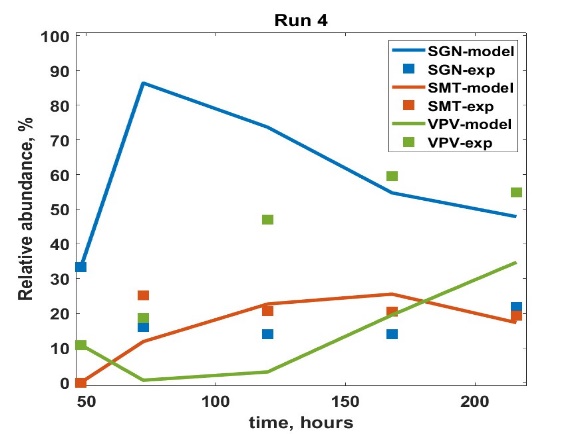** |

**Figure S5** **Relative abundance of *S. gordonii* (SGN), *S. mutans*** (SMT) **and *V. parvula* (VPV) in the biofilm**. Simulation results obtained with pH correction for growth. Experimental data for RE 1 (high glucose, high lactic acid), RE 2 (low glucose, high lactic acid) and RE 3 (low glucose, low lactic acid). The simulation results are presented from time = 48 h to correspond with the first experimental point measured. At time = 0, there are three individual of *S. mutans* for the panels A – C, and five for the panels D-F, while the other four species have the relative abundance measured in the day 2 of the experiments reported in Sangha et al (2024).

***Table S4*** The relative abundance (measured by qPCR) of the bacteria on day 2 of the experiments, when *S. mutans* was added.

| Day 2 experimental relative abundance  Bacterial species | RE1 | RE2 | RE3 |
| --- | --- | --- | --- |
| *Streptococcus gordonii* | 85.38% | 29.80% | 33.34% |
| *Streptococcus mutans* | 0% | 0% | 0% |
| *Actinomyces oris* | 0.06% | 0.04% | 2.42% |
| *Neisseria subflava* | 10.37% | 15.12% | 53.39% |
| *Veillonella parvula* | 4.19% | 55.04% | 10.85% |

**References**

1. Heijnen, J. J. and R. Kleerebezem (2010). Bioenergetics of Microbial Growth. In M.C. Flickinger (Ed.) Encyclopedia of Industrial Biotechnology: Bioprocess, Bioseparation and Cell Technology (pp. 594-617). John Wiley & Sons, New York, USA.
2. Ilie, O., M. C. van Loosdrecht and C. Picioreanu (2012). "Mathematical modelling of tooth demineralisation and pH profiles in dental plaque." Journal of Theoretical Biology **309**: 159-175.
3. Kreft, J. U., C. Picioreanu, J. W. Wimpenny and M. C. van Loosdrecht (2001). "Individual-based modelling of biofilms." Microbiology (Reading) **147**(Pt 11): 2897-2912.
4. Perry, R. H. and D. W. Green (2008). Perry's chemical engineers' handbook. New York, New York : McGraw-Hill.
5. Sangha, J. S., P. Barrett, T. P. Curtis, A. Métris, N. S. Jakubovics and I. D. Ofițeru (2024). "Effects of glucose and lactate on *Streptococcus mutans* abundance in a novel multispecies oral biofilm model." Microbiol Spectr **12**(4): e03713-03723.
6. Yaws, C. L. (2009). Transport Properties of Chemicals and Hydrocarbons. Boston,, William Andrew Publishing.
